# The Impact of COVID-19 Pandemic on Corporate Social Responsibility and Job Embeddedness in China

**DOI:** 10.3389/fpsyg.2022.848902

**Published:** 2022-04-15

**Authors:** Tang Meirun, Steven Lockey, John Blenkinsopp, He Yueyong, Ling Ling

**Affiliations:** ^1^School of Management, Guizhou University, Guiyang, China; ^2^Faculty of Business, Economics and Law, School of Business, The University of Queensland, Brisbane, QLD, Australia; ^3^Oslo New University College, Oslo, Norway; ^4^Northumbria University, Newcastle upon Tyne, United Kingdom; ^5^School of Public Administration, Guizhou University, Guiyang, China

**Keywords:** corporate social responsibility, job embeddedness, organizational identification, China, COVID-19

## Abstract

This article aims to investigate the impact of employee perceptions of corporate social responsibility (CSR) on job embeddedness under the drastic circumstances of coronavirus disease 2019 (COVID-19). This study also investigated the role of organizational identification as a psychological mechanism linking employee perceptions of corporate social responsibility (CSR) to job embeddedness. Survey data were collected from 325 employees in banking industry of China and analyzed using partial least squares structural equation modeling (PLS-SEM). Results revealed that CSR to employees and organizational identification were positively and significantly related to job embeddedness, while CSR to customers, CSR to government, and CSR to society did not significantly predict job embeddedness. Organizational identification fully mediated the relationship between CSR to customers, CSR to government, CSR to society and job embeddedness, and partially mediated the relationship between CSR to employees and job embeddedness. The results suggest engaging in CSR activities can lead employees to identify themselves with the organization and enhance their embeddedness. The article concludes with several implications for practice and recommendations for future research.

## Introduction

Corporate social responsibility (CSR), defined as “an organization’s actions and policies that take into account stakeholders expectations and the triple bottom line of economic, social, and environmental performance” (Aguinis, 2011, p. 855), has been extensively researched. Previous research on CSR mainly focused on organizations (or macro level) rather than individuals (or micro level), but more recent studies have shown the importance of considering the psychological micro-foundations of CSR by studying employee-focused micro-CSR (Rupp and Mallory, 2015). The present study is located within this stream of micro-CSR studies and examines how employees’ perceptions of organizational engagement in CSR (henceforth CSR perceptions) impact on individual attitudes and behavior. Growth in the prevalence of CSR and in public expectations that business should make a social contribution (Gaither et al., 2018) has inevitably led to concern that for some firms CSR is less about doing good and more about appearing to do good (Elving, 2013). Employees, as insiders to the firm, are in a position to see whether the firm’s claims about its CSR are consistent with reality. In particular, they will be the first to notice if a firm’s commitment to CSR weakens when the business environment gets tougher.

The COVID-19 pandemic has created immense and unprecedented pressures on business, with the survival of the firm being on the line in many cases. Under these conditions a firm’s commitment to CSR will be tested, and employees’ CSR perceptions are likely to be sharpened (positively or negatively, depending on how the firm acts). The pandemic has thus created organizational conditions in which it becomes easier to study the underlying relationship between CSR perceptions and key employee outcomes. Previous studies have explored the impact of CSR perceptions on affective commitment (George et al., 2020), performance and organizational pride (Asante Boadi et al., 2020), and work engagement and turnover intention (Lin and Liu, 2017). The present study investigates the effect of CSR perceptions on two other key employee outcomes, organizational identification and job embeddedness, and proposes an underlying mechanism by which CSR perceptions influence employee outcomes.

The COVID-19 pandemic greatly disrupted the global economy, hitting profits and profitability on a scale rarely seen outside of major recessions. This exceptionally difficult time for businesses has put their commitment to ethical business conduct and corporate social responsibility (CSR) to the test (He and Harris, 2020), posing challenges to firms with regard to the struggle of balancing the interest between stakeholders and their own business (Antwi et al., 2021). Under these exceptional pressures we might expect that some firms would be likely to pursue short-term gains and reduce long-term CSR investment due to lack of slack resources and mounting pressure for survival which are caused by the outbreak of COVID-19. However, the pandemic also offers an opportunity for businesses to shift towards more genuine and authentic CSR and contribute to addressing urgent global social and environmental challenges (He and Harris, 2020; Mahmud et al., 2021; Qiu et al., 2021). This potential conflict in priorities provides the backdrop to the current study to investigate the impact of COVID-19 on CSR investment and the response of individuals’ attitudes and behavior in the workplace.

To cope with massive profit losses during the COVID-19 pandemic many business are adopting various downsizing strategies, among them layoff is one of the strategies. As a result a large number of jobs disappeared from the labor market, which has created uncertainty and unrest among the employees of organizations. Accordingly, organizational life in the drastic circumstances of COVID-19 pandemic had never been more challenging. Defined as ‘the combined forces that keep a person from leaving his or her job’ (Yao et al., 2004, p. 159), job embeddedness has been found to positively influence important work-related behaviors such as job performance (Ali et al., 2021), initiative (Teng et al., 2021), work engagement (Ampofo et al., 2021) and innovative behavior (Amankwaa et al., 2021). Increasing the level of embeddedness might thus help employees use their abilities to avoid layoff and promote their retention, and help the companies get through the difficulties of the COVID-19 pandemic. Although some researchers have investigated the antecedents of job embeddedness, such as job characteristics (Rahimnia et al., 2019), job flexibility (Chan et al., 2019), employee advocacy and perceived organizational support (Akgunduz and Sanli, 2016), more work is needed. Bambacas and Kulik (2012) called for investigation into how HR practices impact job embeddedness, and the present study responds to this call, consistent with the idea that CSR and HRM are strongly linked. We examine the impact of employees’ perception of CSR on their job embeddedness, testing the role of organizational identification as the mediating psychological mechanism by which CSR impacts job embeddedness.

Consistent with the emphasis on different stakeholders, CSR can be viewed as having different facets relating to the target stakeholder. Within the present study, we consider CSR towards employees, customers, society, and government (Turker, 2009b). Ghosh and Gurunathan (2014) found employee perceptions of CSR to society and to customers positively influenced employees’ job embeddedness, which in turn had a negative relationship with intention to quit. However, in considering job embeddedness as a mediating variable, those authors did not consider any psychological mechanisms underpinning the relationship between CSR and job embeddedness. More generally, there has been a lack of investigation of such mechanisms within the job embeddedness literature. The present study contributes to addressing this gap.

Drawing on social identity theory, we posit organizational identification to be a key psychological mechanism underpinning the relationship between CSR and job embeddedness. proposed that the groups to which we belong are a vital source of pride and belonging, and the organization in which we are employed represents a particularly salient group (Ashforth and Mael, 1989). As such, we expect that if employees perceive their organization to demonstrate CSR, they will identify with the organization, feel a greater sense of belonging within it, and as a result, become more embedded in it. There has been a lack of empirical examination of such a psychological process between job embeddedness and its antecedents. A meta-analysis and theoretical extension of job embeddedness by Kiazad et al. (2015) included organizational antecedents related to high-performance work practices and non-organizational characteristics that supply embedding resources (e.g., nearby extended family and community attributes), but did not include any psychological constructs in their analysis. As such, by measuring organizational identification and job embeddedness in a Chinese context, the current study builds on this work.

The predictive validity of job embeddedness has been demonstrated across cultures (Ramesh and Gelfand, 2010; Lee et al., 2014; Hom et al., 2017; Nguyen et al., 2017). However, further examination in a broader range of national, cultural, and organizational contexts is needed (Zhang et al., 2012; Lee et al., 2014; Kiazad et al., 2015; Ampofo et al., 2017). In particular, few studies have used samples of employees in China (Hom et al., 2009; Sun et al., 2012; Zhao et al., 2013), a gap which the present study seeks to address.

**Figure 1:**
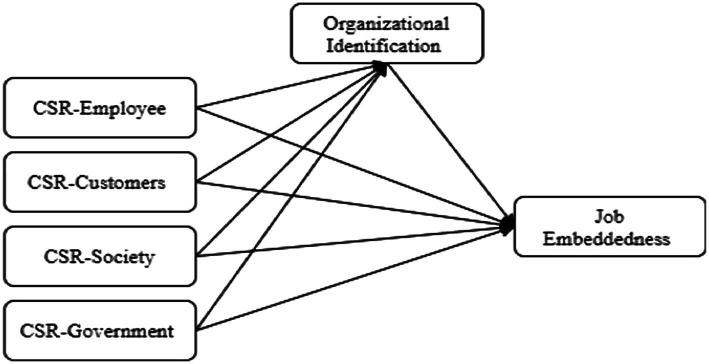
Research framework.

The paper is structured as follows. We begin with reviewing the relevant literature relating to job embeddedness, CSR, and organizational identification, and formulate hypotheses regarding the relationships between these constructs. After outlining the methodology, we present results pertaining to our hypothesized model (as shown in Figure 1), demonstrating that organizational identification fully mediates the relation between CSR to government, CSR to society and job embeddedness respectively, and partially mediates the relationship between CSR to employees and job embeddedness. We conclude with a general discussion and implications for research and practice.

## Literature Review

CSR requires corporates to conduct their managerial strategies in line with the triple bottom line (economic, social, and environmental goals; McLennan and Banks, 2019). Previous studies have provided evidence that successful CSR activities can lead to better firm financial performance and increase firm value (Schaltegger et al., 2019; Hameed et al., 2020). CSR research has been strongly informed by *stakeholder theory*, which proposes that considering the values of other stakeholders is a necessary and implicit requirement of doing business (Freeman, 1994; Freeman et al., 2004; Du et al., 2010; Niazi et al., 2012). The various stakeholders have different needs and expectations of the firm, which means CSR can be thought of in terms of different dimensions relating to distinct stakeholder groups. The extent to which an organization acknowledges its responsibility to society is determined by the ways in which the CSR strategies of the organization define and engage stakeholders (Hameed et al., 2019; Qiu et al., 2021). The diversity of CSR definitions reflect different types of stakeholder groups. The present study adopts the definition of CSR by Turker (2009b), which proposes that CSR include four dimensions, namely CSR to employees, CSR to society, CSR to customers, and CSR to government. We define these dimensions and hypothesize their relations to job embeddedness and organizational identification in the following sections.

### CSR and Job Embeddedness

Previous studies have shown firms generally benefit from their commitment to CSR initiatives. In terms of HR-related benefits, a firm’s CSR initiatives can reduce employee turnover intentions (Lin and Liu, 2017) and counterproductive behaviors (Flammer and Luo, 2017), enhance employee performance and organizational pride (Asante Boadi et al., 2020), and positively impact employees’ work-related attitudes, such as organizational trust and commitment (George et al., 2020). Job embeddedness would seem another outcome that might be influenced by firms’ CSR initiatives but to date few studies have examined this potential link (Ghosh and Gurunathan, 2014).

CSR to employees includes “*good working conditions, including career opportunities, organizational justice, or family-friendly policies*” (Turker, 2009a, p. 192). We posit that a company’s engagement in CSR to employees will affect employees’ embeddedness in the organization. notes that employee-focused CSR activities (such as providing autonomy, avoiding layoffs, and providing life insurance) will positively affect employees. A good internal working environment indicates to staff that the company fulfills the informal contract between itself and employees, which may enhance employees’ psychological attachment, and lead to a stronger perception of fit between the individuals and the organization. The higher likelihood an individual perceives their values or norms to fit with the organization, the more they connect to the organization, and thus they will find it difficult to leave the organization due to the high perceived costs of giving up the benefits that it provides. Prior studies have demonstrated the positive relationship between CSR and organizational commitment (George et al., 2020), and that HR practices such as employee development and rewards are positively related to an individual’s job embeddedness (Bambacas and Kulik, 2012; Van Dyk et al., 2013). We therefore propose the following hypothesis:

*H1a. CSR to employees is positively related to job embeddedness.*

Customers are one of the most crucial stakeholder groups to a firm, so CSR activities to customers are “*a significant tool of influencing the feelings, thoughts, and consequently buying behaviors of their target customers*” (Turker, 2009a, p. 192). Firms’ social performance in providing high-quality service and products or accurate information of their activities sends signals to employees that their employer is proactive and market-oriented (Maignan et al., 1999), making them more likely to remain loyal for reasons of prestige and job security. A firm’s engagement in market-oriented activities indeed leads to proactive corporate citizenship, which in turn is associated with increased customer loyalty, business performance, and employee commitment (Maignan et al., 1999). Conversely, if employees perceive their organization treats customers poorly or does not care about the quality of the goods and services they offer, this may lead them to conclude the organization will not hesitate to keep them in similarly low regard should the need arise. Providing direct support for the importance of customer-related CSR, Ghosh and Gurunathan (2014) found that CSR to customers was positively associated with organization embeddedness in the context of Indian financial managers. Therefore, we propose the following hypothesis:

*H1b. CSR to customers is positively related to job embeddedness.*

CSR to society includes activities aimed at benefiting the “*natural environment, next generations, and non-governmental organizations*” (Turker, 2009a, p. 191), while CSR to government emphasizes legal responsibilities such as *“paying taxes on a regular and continuing basis, and complying with legal regulations completely and promptly”* (Turker, 2009b, p. 422). CSR effects on society and government can create positive associations among employees that affect how they become embedded in their organization. We expect if employees perceive their organization to engage with these types of CSR activities, they will feel the company has good values and norms. In turn, this may create a fit between the employees and their organization (Glavas, 2016). Empirical support for a link between CSR to society and job embeddedness is provided by a study conducted in India (Ghosh and Gurunathan, 2014). Turker (2009b, p. 424) noted that CSR to government had the weakest explanatory power of the four dimensions of his CSR structure. The existence of a legal dimension within such a structure is a contentious issue, but Turker included the dimension in order to “avoid narrowing the concept”. The Chinese context for the present study is one in which this dimension of CSR may prove to be a stronger predictor of employee attitudes than in some countries given the unique role the Chinese government plays in the adoption of CSR (Moon et al., 2010; Moon and Shen, 2010). Overall we propose that:

*H1c. CSR to society is positively related to job embeddednes*s.

*H1d. CSR to government is positively related to job embeddedness.*

### CSR and Organizational Identification

Employee perceptions of their employer’s CSR activities will shape their perceptions of the kind of organization for which they are working. In this section, we consider how each of the CSR dimensions proposed by Turker (2009b) might be expected to impact organizational identification. We start with CSR to employees. An organization’s CSR initiatives that benefit employees can play a significant role in shaping organizational identification. Employees tend to identify with their organization when they believe that their employer perceives them as prominent organizational members and respects them (Tyler and Blader, 2003). The perception of respect enhances the individual’s self-evaluation and shapes their organization-based self-esteem, their belief they are a competent, valuable member of the organization (Pierce and Gardner, 2004). We therefore propose the following hypothesis:

*H2a. There is a positive relationship between CSR to employees and organizational identification*.

Turker (2009a) suggests “positive feedback received from satisfied customers is one of the most effective ways of measuring organizational success.” A firm’s CSR initiatives towards customers may enhance employees’ perceptions of a firm’s corporate attractiveness and reputation, strengthening their positive attitudes to the organization. On the one hand, a firm’s desirable CSR engagement with customers may create the impression to employees that the firm’s characteristics are attractive, which in turn intensifies employees’ identification with the organization. On the other hand, employees may form their identification with the organization when they believe that their firm has a positive reputation among customers. For example, employees may feel proud of their organization if the firm provides high-quality services and products. Conversely, they may feel guilt or shame in the wake of service or product failure (Gillespie et al., 2014). Prior studies have offered significant insights into the role of CSR initiatives in generating organizational identification (Berger et al., 2006; Rodrigo and Arenas, 2008), though they did not examine the customer-focused dimension of CSR to organizational identification.

*H2b. There is a positive relationship between CSR to customers and organizational identification*.

Firms’ CSR activities to society and government may also strengthen employees’ organizational identification. For example, corporate volunteer programs have various benefits concerning employees’ identification processes, employees experience higher morale and self-esteem when participating in corporate volunteer programs. Corporate activities in protecting and improving the quality of the natural environment, considering future generations, or complying with legal regulations completely and promptly can also increase brand reputation and profitability (Khojastehpour and Johns, 2014). The higher the perception of the organization’s prestige, the more likely employees will strongly identify with their organization (Smidts et al., 2001). Membership of a prestigious, respected organization can drive employees’ perceptions of organizational pride (Tyler and Blader, 2003; Fuller et al., 2006). Therefore, employees are more likely to display reliable identification with their organizations, which hold a favorable external reputation and are considered prestigious. This leads to the following hypotheses:

*H2c. There is a positive relationship between CSR to society and organizational identification*.

*H2d. There is a positive relationship between CSR to government and organizational identification*.

### Organizational Identification and Job Embeddedness

Employees with high identification in the organization become psychologically intertwined with it, value it, attach emotional significance to their membership of the organization (Riketta, 2005), and are more likely to co-operate and interact with their co-workers (Dukerich et al., 2002; Islam et al., 2021). This would further prompt them to create “links” within the organization. In addition, identification with the organization reinforces the relationship between “socialization” and “internalization” and facilitates loyalty and commitment to the company, which enables the internalization of organizational values and beliefs (Ashforth and Mael, 1989). Hence, individuals with higher levels of organizational identification might better fit with their organization. Stronger organizational identification is likely to increase the links, fit and sacrifice components that comprise the organizational dimensions of job embeddedness. Research on this linkage has been limited. A study by Jones (2010) found that due to their organizational identification, employees are more likely to stay with the firm. Jones’ findings give us further cause to believe organizational identification should lead to greater embeddedness within the organization, which leads to the following hypotheses:

*H3. There is a positive relationship between organizational identification and job embeddedness*.

### The Mediation Effects of Organizational Identification

Drawing on social identity theory, this study proposes that organizational identification mediates relationships between the dimensions of CSR and job embeddedness. Social identity theory proposes that individuals tend to achieve their salient identity through categorizing themselves into different groups (Fuller et al., 2006), and compare themselves with different members within the same group. These inter-group comparisons further drive them to create uniqueness or status and finally affect their self-esteem (Ashforth and Mael, 1989), self-image within the group, and then strengthen their identification with the organization.

Firms’ CSR activities to employees, customers, society and government are salient source of social identity, which individuals may use to assess self-worth. Employees’ perception of their employers’ CSR initiatives may effectively promote them in categorizing themselves into the organization and perceive similarities between their self-concept and their organizational identity. The more an employee’s perception of their organization’s commitment to employees, customer, society, and government related CSR initiatives, the more the organization’s values, norms, and interests are incorporated into their self-concept, and occurs organizational identification. Employees with high organizational identification become highly psychologically intertwined with the organization, valuing and developing an emotional attachment with it. In turn, these psychological processes inform employees’ attitudes and behaviors, such as job embeddedness.

We therefore propose the following hypothesis:

*H4. Organizational identification mediates relationships between CSR and (a) employees, (b) customers, (c) society, and (d) government and job embeddedness*.

## Methodology

### Sampling, Respondents, and Data Collection

The study was undertaken with employees within the banking industries of China. The sector is currently under stress due to high levels of credit losses from large-scale insolvency in both corporates and households due to the global economic downturn caused by the COVID-19 crisis. Over 500 million people work in this sector and the outbreak of COVID-19 has increased pressure on employees’ performance, leading to high talent turnover. The turnover rate of financial employees prior to the COVID-19 pandemic was 5% but rose to more than 15% after the start of the pandemic (Ma et al., 2021). Managers in the sector thus face a challenge in dealing with these labor turnover problems and so organizations were unsurprisingly open to giving access for a study examining factors that might enhance job embeddedness.

Given the size of the sector it is impossible to get a complete sampling frame. As such, a judgment sampling technique is used to determine the bank location and then draw the final sample from the population. Fujian Province, Guangdong Province, Zhejiang Province, Jiansu Province, and Guizhou Province have been selected because they represent the provinces with high, middle and low Gross Domestic Product (GDP) contribution in China. For the sample size, this study applies G*Power techniques to determine the minimum sample size, and the sampling table to define the maximum sample size. Therefore, a sample size which is between 138 and 384 is the proper sample size for this study.

With the help of Alumni Association from the Guizhou University School of Management, the present study selected five banks from the targeted Provinces. These five banks are selected because their main managers are studying the MBA program in the school of management Guizhou University at that time, and these managers are more likely to help the data collection procedure. A dual-language (English and Chinese version) of the questionnaire was sent, followed up by calls and a cover letter explaining the importance of the study and assurances of utmost confidentiality for participants. The current study distributed 100 surveys to the managers in each of the banks during March 2021, meaning a total of 500 questionnaires were delivered.

The managers in each bank then asked the executives in human resource department to distribute the questionnaires to their employees. To reduce the risk of common method variance, measures were administered across surveys delivered at two-time points. Respondents were asked to rate their perceptions of their organization’s CSR activities at time 1. One month later, the respondents were asked to rate their organizational identification and job embeddedness. Finally, 325 completed surveys were returned, indicating a response rate of 65%. In the returned sample, 55.4% of respondents were male, and 44.6% were female. Regarding age, 28% of the sample was between 18 and 27 years old, 61.8% were between 28 and 37 years old, and 9.2% were between 38 and 47 years old. In terms of education, 76.6% held a bachelor’s degree, and 23.4% of the sample were educated at master level or higher. 59.7% of respondents had an organizational tenure of more than 3 years, while 40.3% worked in their current company for less than 3 years.

### Measurement

Items were rated on a Likert scale from 1 *(strongly disagree)* to 7 *(strongly agree)*. The scales were initially written in English, and the multistage translation model was used to translate them into Chinese.

#### Job Embeddedness

To measure job embeddedness, we used seven items from Crossley et al. (2007). Sample items are “I feel attached to this organization” and “I am tightly connected to this organization.” The survey is much shorter than the composite measure but has a strong positive correlation and construct validity.

#### Corporate Social Responsibility

CSR measures were taken from the CSR Scale of Turker’s (2009b). CSR to employees contains five items, for Example, “our company implements flexible policies to provide Good work and life balance for its employees.” CSR to society comprises seven items, for example, “our company invests in creating a better life for the future generations.” CSR to customers consists of three items, with a sample being: “our company respects consumer rights beyond legal requirements.” CSR to government contains four items, such as “our company always pays its taxes on a regular and continuing basis.”

#### Organizational Identification

Organizational identification was measured using the Mael and Ashforth (1992) six-item organizational identification scale. sample items are “when someone criticizes my company, it feels like a personal insult” and “if a story in the media criticized the company, I would feel embarrassed.”

### Data Analysis

To analyze the data we used partial least squares structural equation model (PLS-SEM), a variance-based structural equation modeling technique to examine the proposed theoretical model (Hair et al., 2016). The PLS-SEM approach is a bootstrapping and blindfolding procedure that maximizes the predictive accuracy of endogenous variables while allowing the retention of more indicators for each construct. This technique is selected for two reasons. First, PLS-SEM does not require data with a normal distribution (Hair et al., 2016, p. 36). In the current study, Mardia’s test of multivariate skewness and kurtosis is below the threshold value of 0.05 suggested by Cain et al. (2017), indicating data are not normally distributed. Second, PLS-SEM is adequate for predictive purposes, which is consistent with the research objective of the current study. Therefore, the use of PLS-SEM was considered an appropriate approach for data analysis. A measurement model and the structural model were assessed in this study.

### Assessment of Measurement Model

To assess the measurement model, internal consistency, indicator reliability, convergent validity, and discriminant validity of the constructs used in this study were examined (see Table 1). All constructs had cronbach alpha (CA) and composite reliability (CR) values higher than the recommended criterion of 0.70 for CA (George and Mallery, 2003) and CR (Nunnally and Bernstein, 1994), and thus achieved internal consistency. Additionally, the indicator loadings of each construct showed all items’ loadings were higher than the suggested cut-off value of 0.70 apart from item JE6, which had a loading of 0.62. Hair et al. (2016) have stated that indicators with outer loadings between 0.40 and 0.70 should be considered for removal from the scale only when deleting the indicators leads to an increase of composite reliability above the suggested threshold value. However, in the current study, the CR value of job embeddedness, at 0.95, has reached the threshold value (see Table 1). Hence, we retain item JE6. Thus, all the indicators in this study have achieved reliability in their corresponding constructs. Furthermore, we examined the average variance extracted (AVE) to assess convergent validity. Table 1 shows that the AVE values of all of the constructs in this study surpass the criterion of 0.50 (Fornell and Larcker, 1981), demonstrating good convergent validity.

**Table 1:** Findings of the measurement model (reflective).

Constructs	Item	Loadings	CA	CR	AVE
CSR-C	CSR-C1	0.88	0.84	0.90	0.76
	CSR-C2	0.90			
	CSR-C3	0.83			
CSR-E	CSR-E1	0.81	0.90	0.93	0.71
	CSR-E2	0.88			
	CSR-E3	0.83			
	CSR-E4	0.86			
	CSR-E5	0.86			
CSR-G	CSR-G1	0.80	0.87	0.91	0.73
	CSR-G2	0.90			
	CSR-G3	0.88			
	CSR-G4	0.84			
CSR-S	CSR-S1	0.85	0.93	0.94	0.70
	CSR-S2	0.89			
	CSR-S3	0.86			
	CSR-S4	0.87			
	CSR-S5	0.78			
	CSR-S6	0.82			
	CSR-S7	0.80			
OID	OID1	0.85	0.94	0.95	0.77
	OID2	0.86			
	OID3	0.86			
	OID4	0.93			
	OID5	0.93			
	OID6	0.86			
JE	JE1	0.85	0.94	0.95	0.73
	JE2	0.88			
	JE3	0.91			
	JE4	0.93			
	JE5	0.89			
	JE6	0.62			
	JE7	0.87			

*CSR-C: Corporate Social Responsibility to Customers; CSR-E: Corporate Social Responsibility to Employees; CSR-G: Corporate Social Responsibility to Government; CSR-S: Corporate Social Responsibility to Society; OID: Organizational Identification; JE: Job Embeddedness*.

Concerning the constructs’ discriminant validity, we evaluated the Heterotrait-Monotrait ratio (HTMT). The HTMT is the ratio of the between-trait correlations to the within-trait correlations. A disattenuated correlation between two constructs close to the value of 0.90 (Gold et al., 2001) indicates a lack of discriminant validity. Table 2 shows the square root of AVE (diagonal) is higher than the correlations (off-diagonal) for all constructs, and all the values fulfill the criterion of HTMT, being less than the value of 0.9 (Gold et al., 2001). Therefore, all the constructs in the current study have achieved discriminant validity.

**Table 2:** Heterotrait-Monotrait ratio.

	CSR-C	CSR-E	CSR-G	CSR-S	JE	OID
CSR-C	**---**	---	---	---	---	---
CSR-E	0.83	**---**	---	---	---	---
CSR-G	0.71	0.63	**---**	---	---	---
CSR-S	0.76	0.81	0.67	**---**	---	---
JE	0.52	0.66	0.46	0.56	**---**	---
OID	0.68	0.67	0.67	0.70	0.73	**---**

*CSR-C: Corporate Social Responsibility to Customers; CSR-E: Corporate Social Responsibility to Employees; CSR-G: Corporate Social Responsibility to Government; CSR-S: Corporate Social Responsibility to Society; OID: Organizational Identification; JE: Job Embeddedness. Numbers parentheses represent the values of Fornell-Larcker; numbers in the table represent the value of Heterotrait-Monotrait Ratio (HTMT)*.

### Assessment of Structural Model

Before commencing hypothesis testing, we assessed the potential collinearity problem by estimating the variance inflation factor (VIF). All the constructs had VIF values less than five indicating collinearity problem between constructs was not a concern (Hair et al., 2011). Thus, further analysis of the structural model can continue. To assess the structural model, Hair et al. (2016) suggested examining the coefficient of determination (*R*^2^ value), beta, and *t*-values through a bootstrapping procedure with 5,000 resamples, and also recommend reporting effect size (ƒ^2^). Thus, by running the bootstrapping with two-tailed tests of a significance level of 5% procedure, this study assessed the structural path model to examine the hypotheses (see Table 3).

**Table 3:** Results of the structural model path coefficient.

Relationships	Std. beta	Std. error	T-value (2-tailed)	*p* (2-tailed)	Decisions	*R* ^*2*^	ƒ^*2*^
CSR-E - > JE	0.39	0.06	6.13	<0.001	Supported	0.56	0.12
CSR-C - > JE	−0.11	0.07	1.63	0.10	Not supported		0.01
CSR-S - > JE	−0.03	0.07	0.43	0.66	Not Supported		<0.01
CSR-G - > JE	−0.06	0.06	1.05	0.29	Not Supported		0.01
OID - > JE	0.59	0.06	10.58	<0.001	Supported		0.36
CSR-E - > OID	0.15	0.07	2.04	0.04	Supported	0.53	0.02
CSR-C - > OID	0.13	0.07	2.04	0.04	Supported		0.02
CSR-S - > OID	0.30	0.07	4.52	<0.001	Supported		0.07
CSR-G - > OID	0.26	0.07	4.02	<0.001	Supported		0.08

*CSR-C: Corporate Social Responsibility to Customers; CSR-E: Corporate Social Responsibility to Employees; CSR-G: Corporate Social Responsibility to Government; CSR-S: Corporate Social Responsibility to Society; OID: Organizational Identification; JE: Job Embeddedness*.

Next, we examined the predictors of job embeddedness. CSR to employees has a significant, positive relationship with job embeddedness (*β* = 0.39, *p* < 0.01), supporting Hypothesis 1a. CSR to customers (*β* = −0.11, *p* > 0.05), CSR to society (*β* = −0.03, *p* > 0.05), and CSR to government (*β* = −0.06, *p* > 0.05) did not show significant relationships with job embeddedness. As such, Hypotheses 1b, c, and d were not supported. Organizational identification was positively related to job embeddedness (*β* = 0.59, *p* < 0.01), supporting Hypothesis 3. These five predictors explain 55.6% of the total variance in job embeddedness. The *R*^2^ value 0.56 is greater than the 0.50 value suggested by Henseler et al. (2009), indicating a moderate model.

We then examined the predictors of organizational identification. CSR to employees (*β* = 0.15, *p* < 0.05), CSR to customers (*β* = 0.13, *p* < 0.05), CSR to society (*β* = 0.30, *p* < 0.01), and CSR to government (*β* = 0.26, *p* < 0.01), were all positively related to organization identification, supporting Hypotheses 2a, b, c, and d. These four predictors explain 53% of the total variance in organizational identification. The *R*^2^ value of 0.53 is higher than the 0.50 value suggested by Henseler et al. (2009), indicating a moderate model.

Furthermore, we also assessed the effect size (ƒ^2^), which is explained as the exogenous variable’s contribution to the *R*^2^ value of the endogenous variable (Chin, 1998). Cohen (1988) suggested that ƒ^2^ effect size values of 0.02, 0.15, and 0.35 represent small, medium, and large effects of exogenous latent variable respectively, while effect size value of less than 0.02 indicates that there is no effect. From Table 3, we found that organizational identification has a large effect size on job embeddedness. CSR to employees has a small-to-medium effect on job embeddedness, CSR to employees, CSR to society, CSR to customers and CSR to government have small effects on organizational identification. The other exogenous variables did not show any effects on either of the endogenous variables.

### Testing Mediating Effects

To investigate mediating effects we used a bias-corrected bootstrap confidence interval approach (Hayes and Scharkow, 2013). Results displayed in Table 4 show the indirect effect of organizational identification on the relationship between CSR to employees and job embeddedness was positive and significant [*β* = 0.09, *p* < 0.05, 95% CI (0.01, 0.17)], supporting Hypothesis 4a. Similar significant results were found for the mediating influence of organizational identification on the relations between CSR to customers and job embeddedness [*β* = 0.08, *p* < 0.05, 95% CI (0.01, 0.16)], CSR to society and job embeddedness [*β* = 0.18, *p* < 0.01, 95% CI (0.09, 0.27)], and CSR to government and job embeddedness [*β* = 0.15, *p* < 0.01, 95% CI (0.07, 0.23)], supporting Hypotheses 4b, c, and d.

**Table 4:** Indirect effects in the mediation model.

Path	Std. beta	Std error	T-values (2-tailed)	*p* (2-tailed)	Confidence interval (BC)		Mediating decision
					LL (2.50%)	UL (97.5%)	
CSR-E - > OID - > JE	0.09	0.04	2.03	0.04	0.01	0.17	Supported
CSR-C - > OID - > JE	0.08	0.04	2.00	0.04	0.01	0.15	Supported
CSR-S - > OID - > JE	0.18	0.04	4.22	<0.001	0.09	0.26	Supported
CSR-G - > OID - > JE	0.15	0.04	3.57	<0.001	0.08	0.24	Supported

*CSR-C: Corporate Social Responsibility to Customers; CSR-E: Corporate Social Responsibility to Employees; CSR-G: Corporate Social Responsibility to Government; CSR-S: Corporate Social Responsibility to Society; OID: Organizational Identification; JE: Job Embeddedness*.

The data allows us to classify the types of mediation. We note that CSR to employees was positively and significantly related to job embeddedness, but CSR to customers, CSR to society, and CSR to government did not have significant relationships with job embeddedness. According to the definition of complementary mediation (the mediation effect and direct effect both exist) and indirect-only mediation (the indirect effect is significant, but the direct effect is not significant; Zhao et al., 2010), we observe that organizational identification plays a complementary mediation role in the relationship between CSR to employees and job embeddedness. We also find that organizational identification has indirect-only mediation among the relationships between CSR to customers and job embeddedness, CSR to society and job embeddedness, CSR to government, and job embeddedness.

## Discussion

The outbreak of COVID-19 posed great challenges for organizations with regard to CSR. Some corporates have tried profiteering from this crisis, while others have proactively become involved in various CSR activities. The COVID-19 pandemic has thus put many corporates under test for commitments to ethical business conduct and CSR (He and Harris, 2020). In the pursuit of social responsibility corporates struggle to balance the interest of society and their own interest. Banking, the sector studied here, offers a good illustration. The COVID-19 pandemic has caused a significant decline in the bank sector of China, and banks are experiencing serious stress due to high levels of credit losses from insolvencies arising from the global economic downturn caused by this crisis. Employees in the bank sectors have never experienced such an uncertain and unsettling situation, when layoff became a key downsizing strategy to cope with losses during the COVID-19 pandemic. Finding ways to increase individuals’ job embeddedness level in the banking sector has never been more challenging.

The current study examined employee attitudes to their organization’s CSR activities and how they impacted job embeddedness directly and *via* organizational identification under the drastic circumstances of COVID-19. Results revealed that CSR to employees was positively related to job embeddedness. The findings of a significant relationship between CSR to employees and job embeddedness indicated that a company’s engagement in CSR to employees could affect an employee’s embeddedness in the organization. This finding is consistent with earlier studies (Bambacas and Kulik, 2012; Van Dyk et al., 2013). However, the results revealed that CSR to customers, CSR to society, and CSR to government, did not directly predict job embeddedness. These nonsignificant relationships were not consistent with Ghosh and Gurunathan’s (2014) findings, which indicated that CSR to customers and CSR to society both positively affected job embeddedness among 501 managers in 19 financial service organizations in India. A potential reason for the significance of “internal” CSR factors over “external” concerns may relate to job-related pressures such as long working hours and high levels of stress prevalent in Chinese financial organizations. Despite China’s labor law stating that workers should not work more than 44 hours a week, a recent report suggests that just under half of the financial employees surveyed work from 46 to 55 h per week, almost 40% work from 55 to 65 h per week, and just over 10% work more than 65 h per week. The same report suggests that employees in the Chinese banking industry experience more health problems than workers in other sectors.

The resource strain of working under such conditions may explain why employees prize CSR activities that alleviate the pressure on them over externally-focused activities that do not directly benefit them. Conservation of resources theory (CoR) may provide some theoretical underpinning for this proposition. The central tenet of CoR is that people strive to develop, maintain, and protect valued resources and will engage with and value activities that support these goals. Resources are “*those objects, personal characteristics, conditions, or energies that are valued by the individual or that serve as a means for the attainment of those objects, personal characteristics, conditions or energies*”. As such, CSR activities that alleviate stress and workload issues can be seen as resources that energize employees and embed them in their jobs. Conversely, external CSR activities may be less valuable to employees and be less embedding, particularly if their resource pools are already low due to high levels of strain caused by long working hours and high levels of stress.

Mediation analyses indicated that organizational identification partially mediates the relationship between CSR to employees and job embeddedness and fully mediates the relations between CSR to customers and job embeddedness, CSR to society and job embeddedness, and CSR to government and job embeddedness. These results show perceptions of CSR to employees, CSR to society, and CSR to government, could facilitate employees’ identification with their organization, and in turn, increase their embeddedness in their job. These findings provide empirical evidence indicating the importance of organizational identification as an underpinning mechanism linking CSR to job embeddedness.

### Theoretical Implications

Previous studies of CSR are mainly focused on macro level rather than micro level. Our study contributes to the emerging literature aimed at addressing this gap by extending the studies of CSR from an organizational-focused macro perspective to an employee-focused micro perspective. This study is located in the employee-focused research stream and examines how employees’ perceptions of organizational involvement in CSR affect individual attitudes and behaviors. This is one of the first studies that examined the combined effects of CSR to employees, CSR to customers, CSR to society, CSR to government, and organizational identification on job embeddedness in the Chinese context and is particularly novel in testing these relationships under the drastic circumstances of COVID-19.

The current study contributes to the existing literature in three ways. First, the empirical results contribute to theoretical understanding of job embeddedness. There are many studies have established the link between various antecedents and job embeddedness, but very few focus on the linkage between CSR strategies and job embeddedness. This study enrich the job embeddedness literature by confirming that CSR strategies can served as important embeddedness resource that improve individual retention in the workplace. Our study demonstrated that corporate commitment to employees, customer, society, and government related CSR initiatives can enhance employees’ psychological attachment, such as the perception of fit between the individuals and the organization. The higher likelihood an individual perceives their values or norms fit with the organization, the more they connect to the organization, and thus they will find it difficult to leave the organization due to the high perceived costs of giving up the benefits that it provides. This study is one of the early research that link the relationship between employees’ perception of CSR and job embeddedness.

Second, by applying social identity theory, the current study confirmed that organizational identification is a key psychological mechanism underpinning the relationship between employees’ perception of CSR and job embeddedness. Corporate commitment to employees, customer, society, and government related CSR initiatives can play a critical role in shaping employees’ perceptions of the organization for which they are working. Employees tend to identify with organizations that behave responsibly both internally and externally, and this influences the process by which they become embedded in their positions. To our knowledge, this study is one of the early research indicating organizational identification has a mediation effect on the relationship between employees’ perception of CSR and their job embeddedness. Finally, our study responds to calls to extend the research of job embeddedness to a non-western context, showing that this concept developed in the West is relevant and applicable to a non-Western country, in this case, China.

### Practical Implications

More and more businesses recognize that their long-term survival and development hinges on achieving a delicate balance between profitability and harmony with its various stakeholders. They have realized it is not about whether or not to invest in CSR, but more about how to invest in CSR to achieve the mutually beneficial and interdependent social/environmental and economic goals (He and Harris, 2020). The COVID-19 pandemic offered a range of significant opportunities to companies who can act strategically in their approach to CSR.

This study has critical implications for the implementation of CSR strategies during and after the COVID-19 pandemic. First, the positive relationship between four dimensions of employees’ perception of CSR and organizational identification emphasizes the positive impact of investment in CSR activities. Second, the significant and positive association between CSR to employees and job embeddedness adds further evidence for the value employers can gain from investment in good working conditions, including career opportunities, organizational justice, and family-friendly policies. The implementation of ethical intergroup practices to employees can enhance their psychological attachment to the organization and bind them to it. Third, the relationships between CSR to society and job embeddedness, CSR to government and job embeddedness suggest that although the implementation of CSR to the natural environment, next generations, non-governmental organizations, or complying with legal regulations did not directly influence employees’ embeddedness, it indirectly benefits job embeddedness *via* organizational identification. Hence, the company’s commitment to CSR practices should not be restricted to the internal stakeholder of employees but may also reflect external stakeholders of the natural environment, next generations, non-governmental organizations, or complying with legal regulations, especially amid the COVID-19 pandemic. Overall, this study emphasizes the importance organizations under the COVID-19 pandemic should attach to CSR activities in terms of employees, and society, which can trigger employees’ identification in the organization and finally lead to high embeddedness in the jobs, and finally facilitate corporates to survive in this crisis.

## Conclusion

The current study is one of the first to investigate the relationship between CSR to employees, CSR to customers, CSR to society, CSR to government, organizational identification, and job embeddedness under the drastic circumstances of COVID-19. We acknowledge however some limitations which should be borne in mind when developing future research. First, the data collection was limited to the context of the banking industry of China, and further work is needed to confirm these findings generalize to different industries and countries. Second, this study is cross-sectional in design, and a longitudinal study is required to validate the proposed linkages. Finally, as China is a collectivist society, it would be helpful to undertake further comparative research in diverse settings.

Despite these limitations, the study does provide evidence that organizational identification is the psychological mechanism linking employee perceptions of corporate social responsibility (CSR) to job embeddedness. Although CSR to society and CSR to government do not significantly predict job embeddedness directly, they are still significantly related to job embeddedness *via* the mediation effect of organization identification. Employees identify with organizations that behave responsibly internally and externally, establishing embeddedness in their jobs in the process. The findings emphasize the positive impact of banks’ investment in CSR activities in arresting employees’ high turnover in the burgeoning Chinese financial sector.

## Data Availability Statement

The raw data supporting the conclusions of this article will be made available by the authors, without undue reservation.

## Author Contributions

TM: conceptualization, methodology, validation, formal analysis, and writing—original draft preparation. SL and JB: writing—review and editing. HY: investigation and data curation. LL: Data collection and financial sponsor. All authors contributed to the article and approved the submitted version.

## Funding

This study is funded by Guizhou Provincial Education Department Humanities and Social Science Research Project “Country and Regional Research Project”: Study of promotion mechanism and realization path of Chinese private corporates social responsibility ecological governance along the Belt and Road (No: 2022GBzd001) and National Natural Science Foundation of China: Research on the Influence Mechanism of Employability on Turnover Intention of the New Generation Employees and Organization Intervention Strategy (No.71862007).

## Conflict of Interest

The authors declare that the research was conducted in the absence of any commercial or financial relationships that could be construed as a potential conflict of interest.

## Publisher’s Note

All claims expressed in this article are solely those of the authors and do not necessarily represent those of their affiliated organizations, or those of the publisher, the editors and the reviewers. Any product that may be evaluated in this article, or claim that may be made by its manufacturer, is not guaranteed or endorsed by the publisher.
